# Blood transcriptome analysis in a buck-ewe hybrid points towards an nuclear factor-kappa B lymphoproliferative autoimmune disorder

**DOI:** 10.1038/s41598-023-38407-z

**Published:** 2023-07-24

**Authors:** Clemens Falker-Gieske, Jens Tetens

**Affiliations:** 1 Division of Functional Breeding, Department of Animal Sciences, Georg-August-University, Burckhardtweg 2, 37077 Göttingen, Germany; 2 Center for Integrated Breeding Research, Georg-August-University, Albrecht-Thaer-Weg 3, 37075 Göttingen, Germany

**Keywords:** Evolutionary genetics, Computational biology and bioinformatics, Gene expression

## Abstract

Mammal hybridization is a speciation mechanism and an evolutionary driver. Goat-sheep, especially buck-ewe hybrids, are very rare with only one case reported in 2016, which is the subject of the work presented here. Blood transcriptome analysis revealed that the hybrid largely deviated from imprinting schemes previously described in sheep and other mammals. Furthermore, transcriptome regulation seems to differ from the parent transcriptomes, which is most likely a product of partially incompatible imprinting mechanisms from two closely related species. To gain a deeper understanding of hybridization in mammals we re-analyzed the RNA sequencing data of the buck-ewe hybrid and its parents. We found parent-of-origin-specific expression of genes that functionally clustered, which we explain with the Dobzhansky–Muller incompatibility (DMI) model. According to the DMI model, proteins which interact have a high probability of being barrier loci and hence are prone to monoallelic expression. We discovered enrichment of genes uniquely expressed by the buck-ewe hybrid, which implicate that it suffered from an NF-κB lymphoproliferative autoimmune disorder. Similar findings were reported in the F1 generation of hybrid mice. We propose that hybridization of two related species may lead to an autoimmune phenotype, due to immunoglobulin incompatibilities and incomplete silencing of barrier loci.

## Introduction

Goat-sheep hybrids (geeps) are rare and most of the described cases were offspring of rams mating with does^1–3^. Hybridization in mammals, however, is a common phenomenon and occurs at so-called hybrid zones, where phylogenetically related species come into contact^4,5^. Most hybrid animals are sterile, which is caused by chromosomal incompatibilities of the parental genomes. This leads to failure of gamete formation during meiosis^6^, which is most likely caused by divergent evolution leading to different structural variation patterns between closely related species^7^. Copy number variants (CNVs) caused by deletions and insertions^8^ as well as inversions^9^ have been discussed to be causative. Other factors preventing gene flow and consequently speciation events are barrier loci^10^. In the special case of goat-sheep hybrid embryos, hemolytic disease caused by an immune reaction of the mother against fetal red blood cells has been identified to prevent the development of hybrid embryos^11^. In the rare case of successful conception by a hybrid, the resulting offspring is considered a new species^12^, which makes hybrid speciation a mechanism of evolution^13^. The study presented here focuses on the analysis of the blood transcriptomes of a geep and its parents. The geep under investigation was born near Göttingen (Lower Saxony, Germany) in March 2014 as the result of the mating between a buck and an ewe. The geep died in 2018 due to polyhydramnios during pregnancy. It was pregnant with two fetuses at advanced developmental stages. The cytogenetics of the geep revealed that it had an intermediate karyotype of 57 chromosomes, whereas the buck had 60 and the ewe 54 chromosomes^14^. An in-depth analysis of the blood transcriptomes of the geep and its parents revealed abnormal imprinting patterns, which we concluded is a compensation mechanism for disadvantageous alleles^15^. Since this study was conducted using reference genome versions, which are now outdated, we re-analyzed the transcriptomes of the geep and its parents and found strong evidence for an NF-κB (nuclear factor 'kappa-light-chain-enhancer' of activated B-cells) autoimmune lymphoproliferative disorder in the geep.

## Results

In this study, the blood transcriptomes of a buck-ewe hybrid and its parents^15^ were re-analyzed to elucidate if the usage of the most recent reference genome assemblies of *Ovis aries* (*O. aries*) and *Capra hircus* (*C. hircus*) reveal new insights to the biology of a mammal hybrid. A prerequisite for the analysis of RNAseq data from a hybrid animal is species discrimination of sequencing reads. To achieve that, the state-of-the-art sequence alignment software HISAT2^16^ was used to map the reads of all three individuals to the *O. aries* and *C. hircus* genomes. The program HyScore, which we developed in our previous study^15^, was applied to the HISAT2 output to assign RNAseq reads unambiguously to one reference genome. This led to an exon coverage of about 18–26 × in all three animals (Table 1).

**Table 1:** Mapping and filtering results of RNAseq data of the buck-ewe hybrid (geep) and its parents.

	*O. aries* (ARS-UI_Ramb_v2.0)	*C. hircus* (ARS1.2)	HyScore *O. aries*	HyScore *C. hircus*	No. of reads	Exon coverage
Geep	68.93%	68.42%	**9.00%**	**8.00%**	**16.4 mio**	21.8 x
Sheep	74.59%	64.34%	**17.00%**	2.00%	**15.5 mio**	18.4 x
Goat	66.14%	77.30%	2.00%	**18.00%**	**16.9 mio**	26.4 x

Results highlighted in bold were used for further analyses.

**Figure 1:**
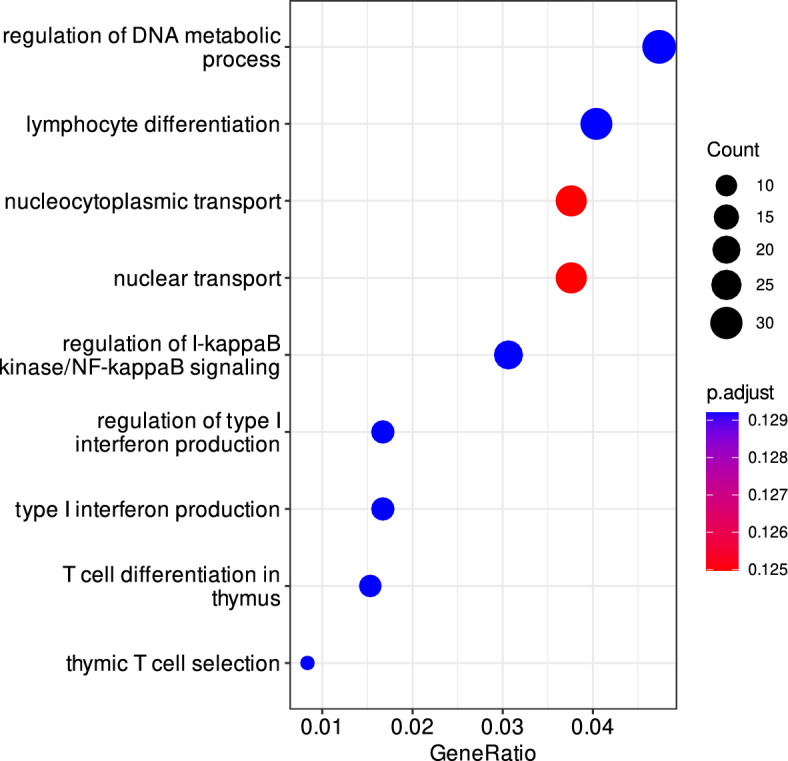
Gene cluster enrichment results for GO biological processes with genes uniquely expressed in the blood of the buck-ewe hybrid.

Genes with a Fragments per kilobase million (FPKM) value > 1 were considered expressed, which led to the discovery of 863 genes expressed in geep blood, which were assigned to the maternal genome and 1100 genes assigned to the paternal genome. In total 659 genes were expressed in the blood of the hybrid but in neither parent blood transcriptomes. These are referred to in the following text as “uniquely expresses geep genes”. Gene cluster enrichment analysis with clusterProfiler revealed a clear differentiation of gene clusters, depending on the parental origin of genes expressed in the blood of the geep (graphical results in Supplementary File S1, tabular results in Supplementary File S2). For instance, biological processes driven by sheep-specific gene expression were ATP and amino acid metabolism, whereas catabolic and cell cycle processes are dominated by expression from the goat’s genome. Regarding cellular components, terms involving proton-transporting ATP synthase complex were enriched in the genes of maternal origin. Biological processes terms related to the degradation of molecules were of paternal origin. Surprisingly, numerous disease-related KEGG pathways were discovered, mostly of maternal origin. These almost exclusively comprised neurodegenerative disorders. The most striking finding, however, was the enrichment of uniquely expressed geep genes for biological processes belonging to the immune system (Fig. 1). These included lymphocyte differentiation, regulation of NF-κB signaling, and type I interferon production.

In a text mining approach with String^17^, we discovered 146 peer-reviewed papers linking 45 genes, which were uniquely expressed in the geep, to autoimmune diseases (Supplementary File S3). Of those studies, 17 focused on the lymphatic system, which is responsible for adaptive immunity. Multiple sclerosis and systemic lupus were detected four times. Numerous genes were detected multiple times (Table 2), with *BCL2, CD28*, and *FASLG* having more than 50 hits.

**Table 2:** Text mining results for genes linked to immune system GO biological processes terms, which were uniquely expressed in the buck-ewe hybrid.

Gene Symbol	Linked to autoimmunity in # publications
BCL2	68
CD28	64
FASLG	53
BCL2L1	47
GATA3	42
IL15	37
GPR29	29
LAG3	28
CHUK	21
TRAF5	17
HIF1A	16
IFIH1	16
NOD2	16
CR2	15
FADD	14
RUNX3	13
TRIM21	12

Peer-reviewed publications listed in NCBI PubMed were mined with STRING (accessed October 2022). The list contains genes linked to autoimmunity by more than 10 peer-reviewed studies.

**Figure 2:**
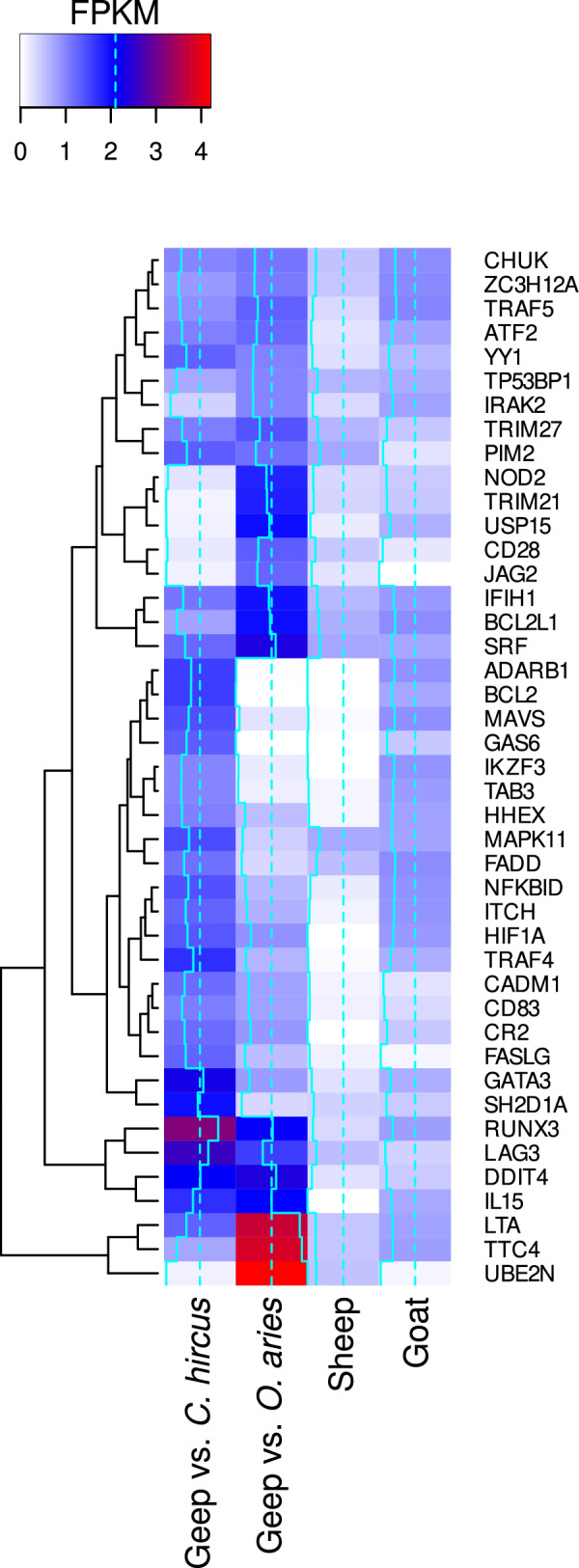
Heatmap of fragments per kilo base million (FPKM) values of genes uniquely expressed in the blood of the buck-ewe hybrid, which were linked to autoimmunity, in comparison to gene expression in its parents. The heatmap was constructed with the Enhanced Heat Map function from the R package gplots (version 3.1.3, https://github.com/talgalili/gplots).

We detected strong expression of *LTA*, *TTC4* and *UBE2N* from the maternal genome and moderate expression of numerous other genes (Fig. 2) involved in autoimmunity, based on “PubMed” enrichment analysis with STRING^17^. Some of those genes were strongly expressed from both parental genomes at similar levels, e.g. *RUNX3*, *LAG3*, *DDIT4*, and *IL15*.

**Figure 3:**
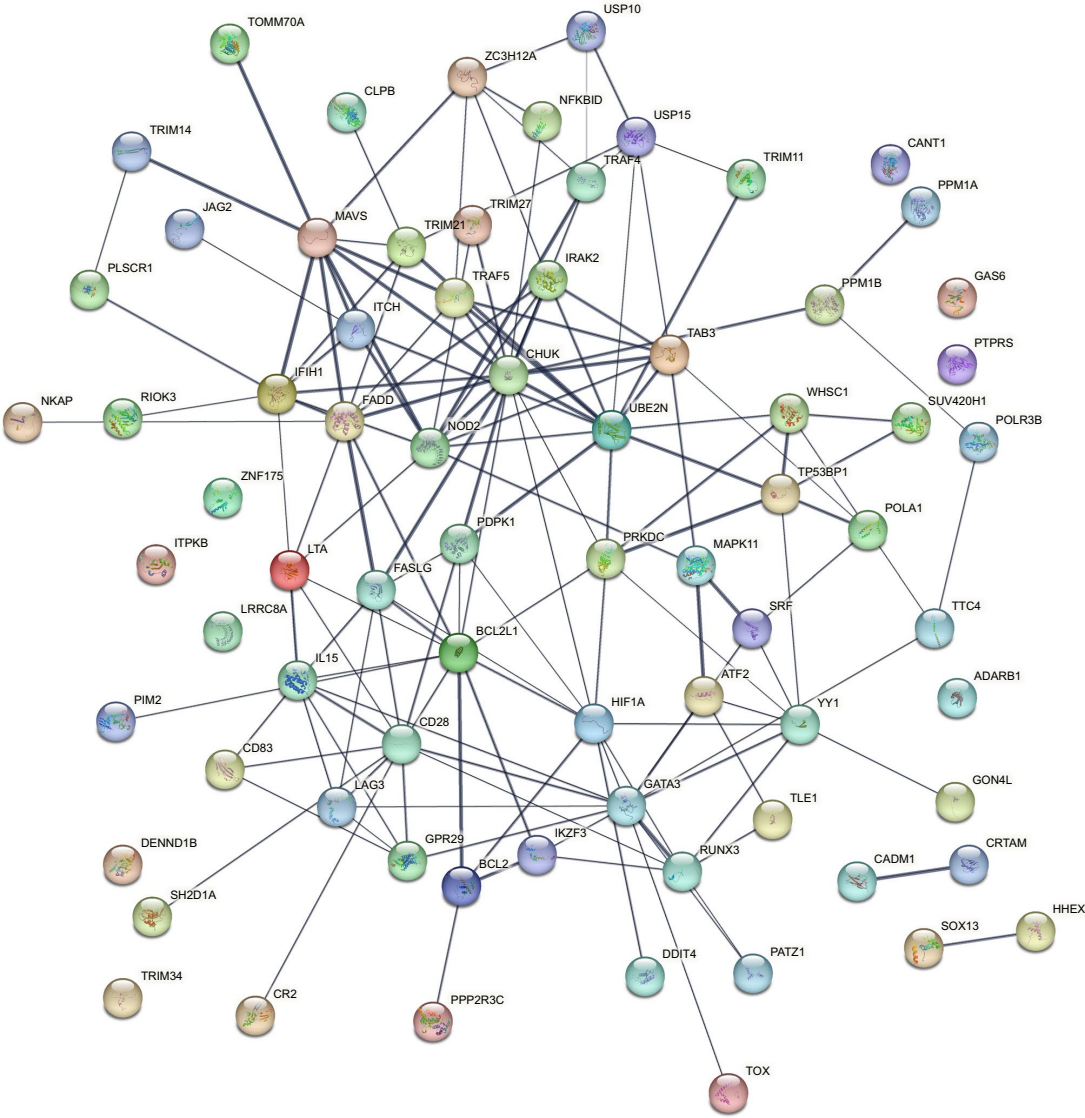
Protein–protein interaction map of genes uniquely expressed in the blood of the buck-ewe hybrid.

Protein–protein interaction (PPI) analysis of uniquely expressed geep genes assigned to immune system GO terms with String^17^ revealed an extensive network of interacting proteins (Fig. 3; PPI enrichment *P* value: < 1 × 10^–16^, number of nodes: 74, number of edges: 154, expected number of edges: 45, average node degree: 4.16, avg. local clustering coefficient: 0.466). *UBE2N*, the gene with the highest expression among unique geep genes, was located at the core of the PPI network connected to multiple interaction partners. Furthermore, CD28, which was linked to autoimmunity in 64 peer-reviewed articles, was strongly interconnected in the PPI network.

## Discussion

One mechanism, which was proposed to explain the reproductive barrier between sheep and goats is hemolytic disease caused by an immune reaction of the mother against fetal red blood cells^11^. The cause of death of the geep was polyhydramnios, a condition often presenting with fetal hemolytic anemia^18,19^. In a recent review Collins et al*.* discussed the function of immunoglobulin genes in reproductive isolation^20^. The authors came to the conclusion that coordination of evolution between heavy- and light-chain genes is crucial to avoid autoimmunity in vertebrate hybrids, which could lead to incompatibilities between hybrids and their offspring. They base their assumptions on the Dobzhansky–Muller incompatibility (DMI) model, which assumes genes encoding proteins that interact, are potential barrier loci because the interaction of gene products between different species would be too low in the hybrid offspring^21,22^. Genes encoding the antibody repertoire of a population change rapidly under selection pressure, which explains their high level of incompatibility between closely related species. Hence, monoallelic expression of genes from one parental genome is a mechanism to overcome these barrier loci. We found evidence for this mechanism in our previous study, where we showed that advantageous alleles were expressed in a monoallelic fashion in the blood of the geep^15^. In the study presented here, we show a clear functional clustering of genes by parental origin, which provides further evidence for the DMI model and as a result a disturbed immune system in the geep. Our results suggest that genes involved in autoimmunity are upregulated in the blood of the buck-ewe hybrid under investigation. This might not only help to understand its unexpected death during pregnancy but may also provide important insight into the immune biology of a mammal hybrid and the consequences on individual fitness. We indeed found several links to hemolytic diseases just by looking at the genes which were uniquely expressed in the blood of the hybrid. *BCL2*, for instance, is a pharmacological target in autoimmune hemolytic anemia (AIHA)^23^. Furthermore, AIHA patients have a significantly higher frequency and absolute count of CD28 null T helper, which also negatively correlates to the hemoglobin levels^24^. *FASLG* was linked to the syndrome of hemolysis, elevated liver enzymes, and low platelets (HELLP)^25^. Pro-inflammatory properties in HELLP were also attributed to *NOD2*^26^.

While this evidence is rather descriptive, a text mining approach led to the identification of a significant number of genes linked to lymphoproliferative and other autoimmune disorders involving lymphocytes. Immunoglobulins are essential for the correct assembly of lymphocytes^27^ and defective antibody gene rearrangements were found in individuals suffering from autoimmune diseases, like systemic lupus^28^. Genes that have been linked to lymphocytic disorders by more than three studies were *BCL2*, *FASLG*, *CD28*, *FADD*, *IL15*, and *BCL2L1*. Another disease captured by text mining was systemic lupus. Symptomatic resemblance to systemic lupus in mice was described over 50 years ago in the F1 hybrids of New Zealand black (NZB) and New Zealand white (NZW) mice^29^. *CD28* was connected to systemic lupus by three studies^30–32^ and *FASLG*^30,32^ as well as TRAF5^31,32^ by two studies. While these lines of evidence put *CD28*, *FASLG*, and *BCL2* into the main focus of an autoimmune phenotype in mammal hybrids, our data suggest that *UBE2N* plays a major role in regulation of the syndrome. With the highest expression among all uniquely expressed genes in the hybrid and its central position in the PPI network (Figs. 2 and 3), it interacts with eight proteins in the map. Yamamoto et al*.* demonstrated that the E2 ubiquitin-conjugating enzyme *UBE2N* (*Ubc13* in mice) tags target proteins, which ultimately results in the activation of the transcription factor NF-κB^33^, one of the top pathways identified by gene cluster enrichment analysis (Fig. 1). Indeed, the direct interaction partners *IRAK2*, *TRAF5*, *CHUK*, and *TAB3* belong to the STRING local network cluster CL:18492 (*false discovery rate* = 0.01, https://string-db.org/cgi/network?taskId=bvmXRveD5alc&sessionId=btqaaLV5T8VE, accessed October 2022), an NF-κB signaling protein interaction cluster.

In summary, the evidence presented here points towards an NF-κB autoimmune disorder involving the lymphatic system in the buck-ewe hybrid, which is most likely caused by the incomplete silencing of barrier loci and immunoglobulin incompatibilities. We assume that the disorder is induced by high *UBE2N* expression, which in turn might lead to ubiquitination of *TAB3*, as outlined by Ruland et al.^34^, and ultimately to the release of NF-κB. NF-κB has been linked to various autoimmune diseases in humans (reviewed by Barnabei et al*.*^35^). Among those is a condition termed *RELA* Haploinsufficiency, which is an autoimmune lymphoproliferative syndrome^36^. Cause of the disease is a de-novo heterozygous nonsense mutation in the *RELA* gene, which encodes the transcription factor p65. It is possible that genomic rearrangements in the buck-ewe hybrid due to chromosomal incompatibility of the parent genomes during meiosis^6^ led to a heterozygous deleterious mutation in RELA or one parental copy has been silenced. However, since the geep was evidently healthy (it was not closely medically monitored), our findings provide rationale for the development of polyhydramnios during pregnancy: an autoimmune phenotype of the hybrid might have been sufficient to lead to an immune reaction against fetal red blood cells. Autoimmune diseases in humans increase miscarriage risks but also reduce female fertility (reviewed by Gleicher et al*.*^37^). We strongly suggest, that this mechanism should be considered in future research focusing on the reproductive barrier between related species. This line of evidence will be subject of future studies, which will focus on the genome assembly of the buck-ewe hybrid, its parents and the fetus.

## Material and methods

### Transcriptome analysis

Reads were aligned to the most recent *O. aries* and *C. hircus* genomes (*O. aries*: ARS-UI_Ramb_v2.0 GCF_016772045.1, *C. hircus*: ARS1.2 GCF_001704415.2) using HiSat2 version 2.1.0 with default settings^16^. Splice sites were derived from the Gene transfer format (GTF) files. Mapped reads were filtered by determining the best alignment result using HyScore^15^. FPKM values were calculated with Cufflinks version 2.2.1^38^. Genes with an FPKM value > 1 were considered expressed. The heatmap was constructed with the Enhanced Heat Map function from the R package gplots (version 3.1.3, https://github.com/talgalili/gplots).

### Functional analyses

Gene cluster comparison and visualization was performed with the R package clusterProfiler (version 4.2.2)^39^. Gene symbols were converted to ensemble IDs with the clusterProfiler Biological Id Translator (bitr) using the org.Hs.eg.db database. GO term analyses were performed with enrichGO (settings: pAdjustMethod = "fdr", pvalueCutoff = 1, qvalueCutoff = 0.25, readable = TRUE, minGSSize = 10). KEGG pathway^40^ analysis was done with enrichKEGG (settings: pvalueCutoff = 1, pAdjustMethod = "BH", minGSSize = 10, maxGSSize = 500, qvalueCutoff = 0.25, use_internal_data = FALSE). Plots were created with the dotplot function. Genes with an enrichment *P* value < 0.001 for immune system related GO biological processes terms were used for downstream analyses. PPI analysis and NCBI PubMed text mining were performed with STRING (version 11.0)^17^ using default settings.

## Supplementary Information

Supplementary Information 1.

Supplementary Information 2.

Supplementary Information 3.

## Data Availability

The raw sequencing data is accessible via BioProject ID PRJNA588993 (https://www.ebi.ac.uk/ena/browser/view/PRJNA588993).
