# Supplementary material for: Blood transcriptome analysis in a buck-ewe hybrid points towards an nuclear factor-kappa B lymphoproliferative autoimmune disorder
Source: Sci Rep. 2023 Jul 24;13:11964. doi: 10.1038/s41598-023-38407-z (PMC10366220; doi:10.1038/s41598-023-38407-z)

Supplementary File S1: Figure of comparative gene cluster enrichment results of buck-ewe hybrid genes expressed from the maternal or the paternal genome.

GO biological processes

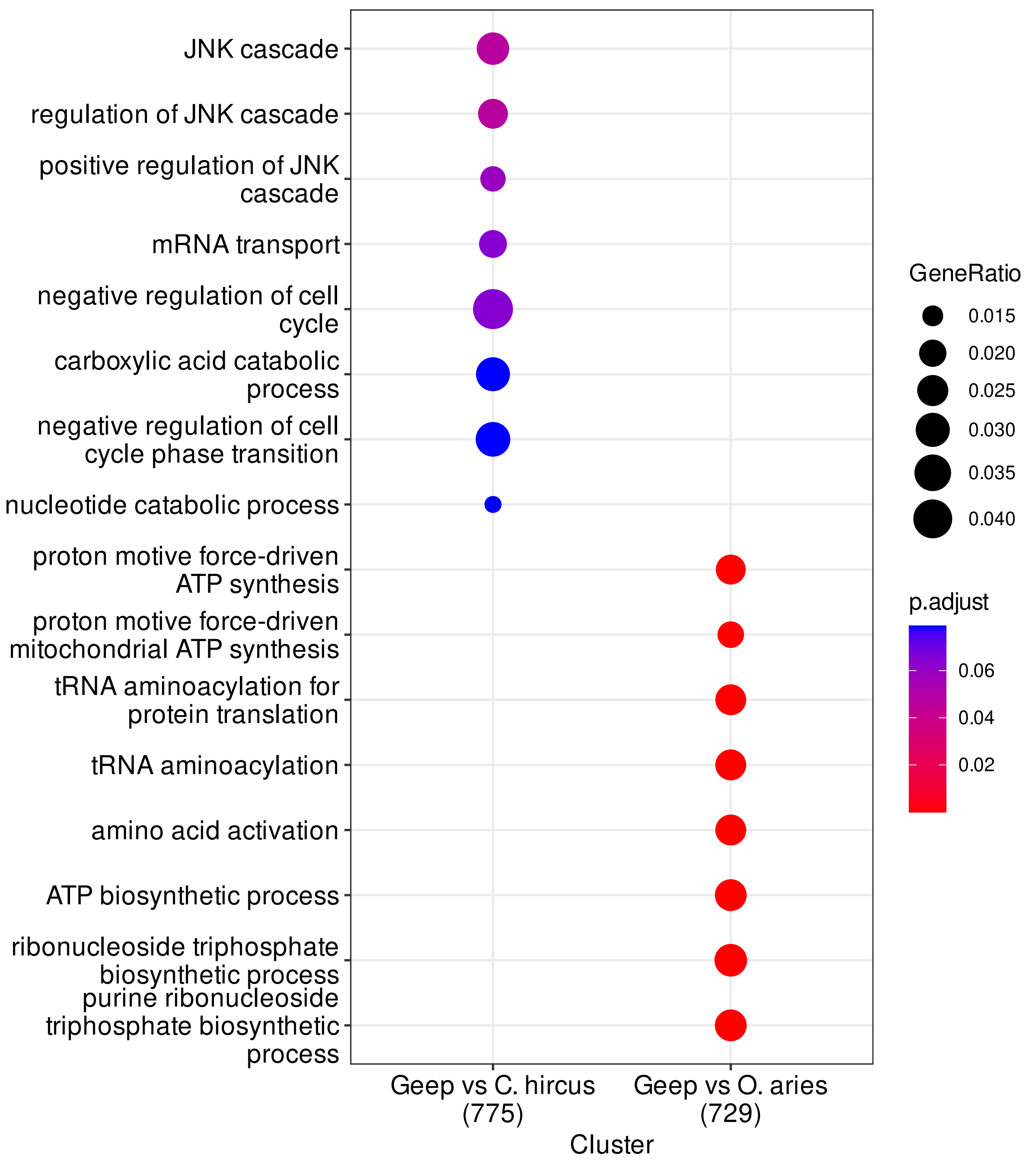

GO cellular component

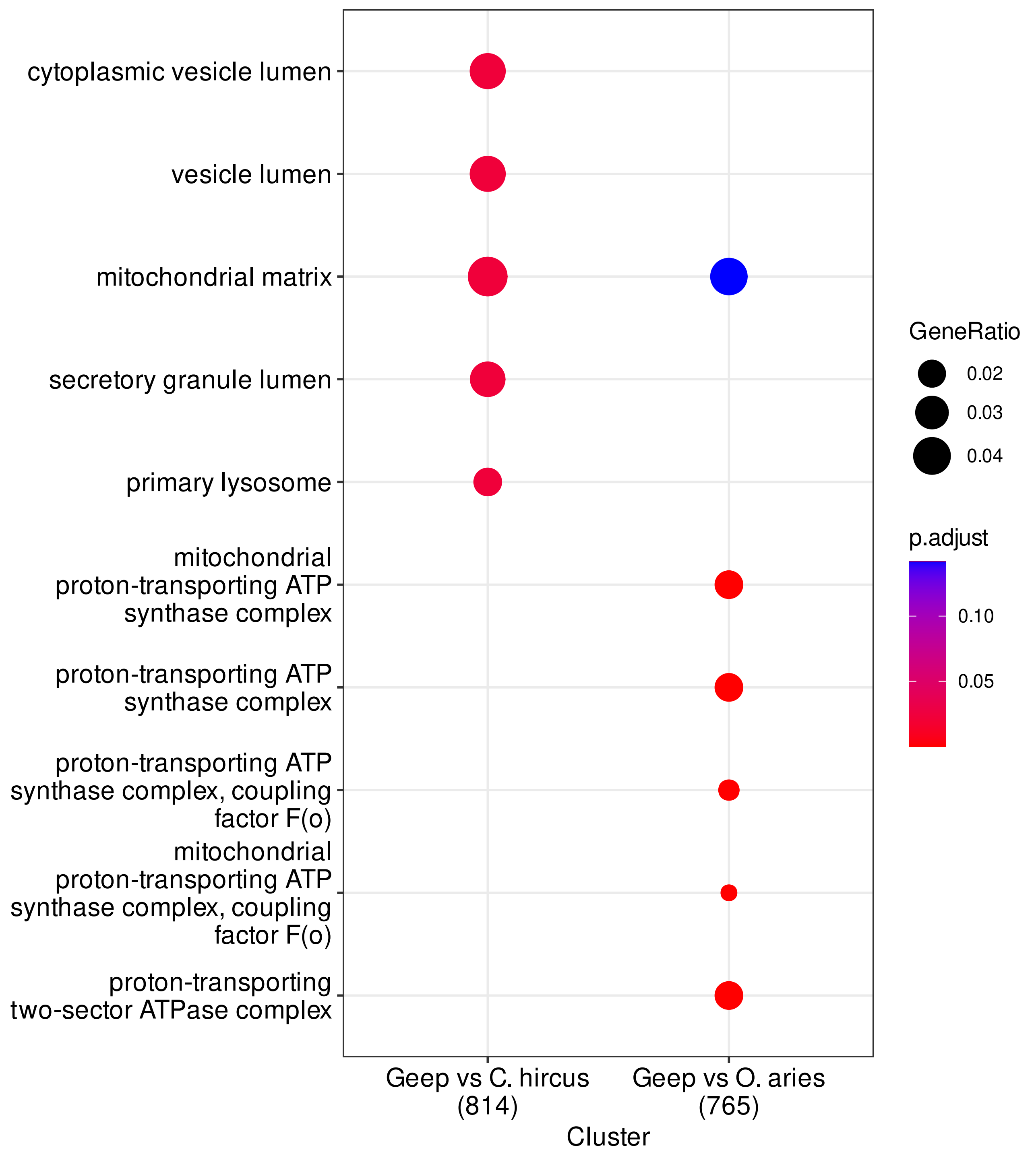

GO molecular functions

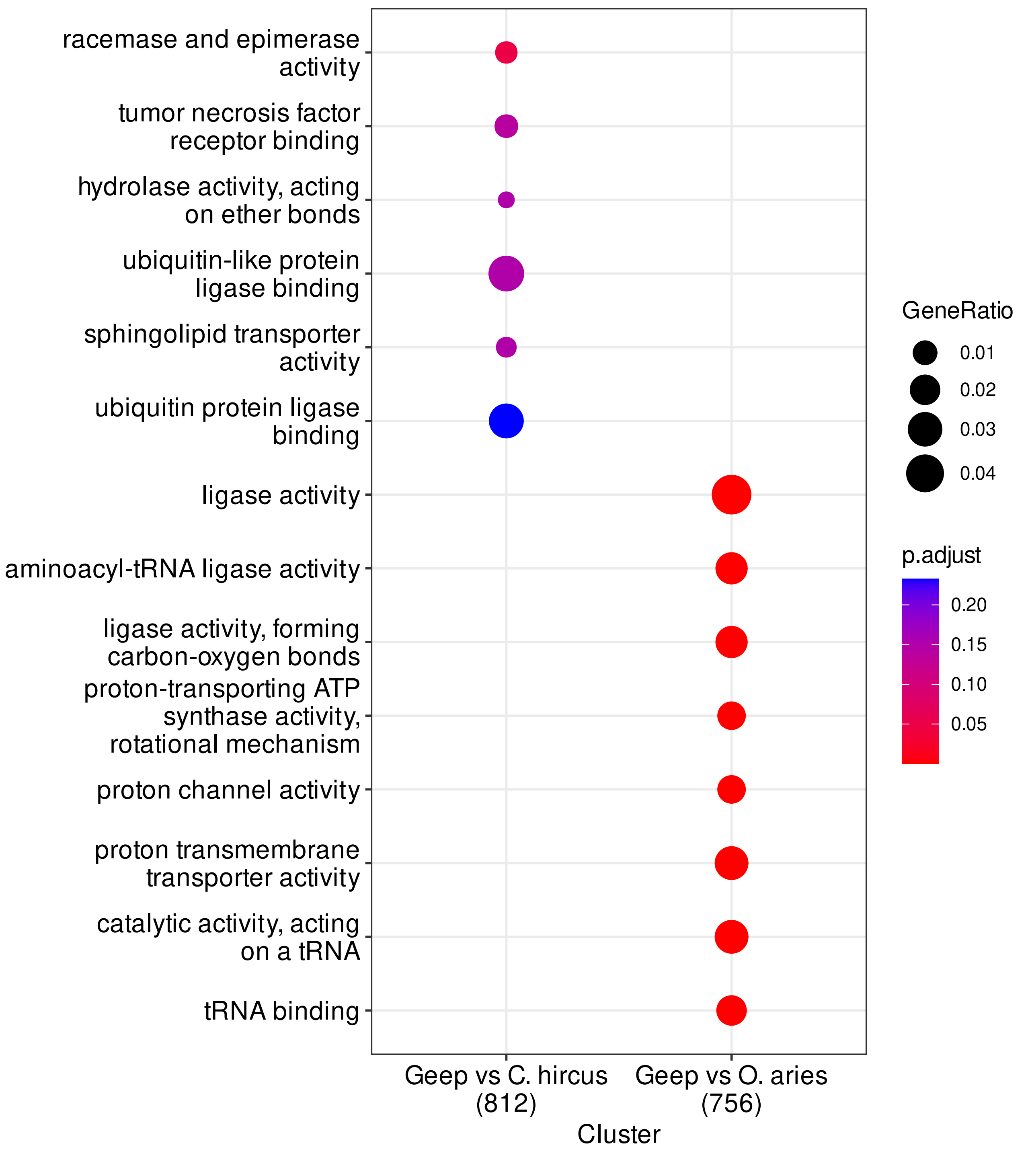

KEGG

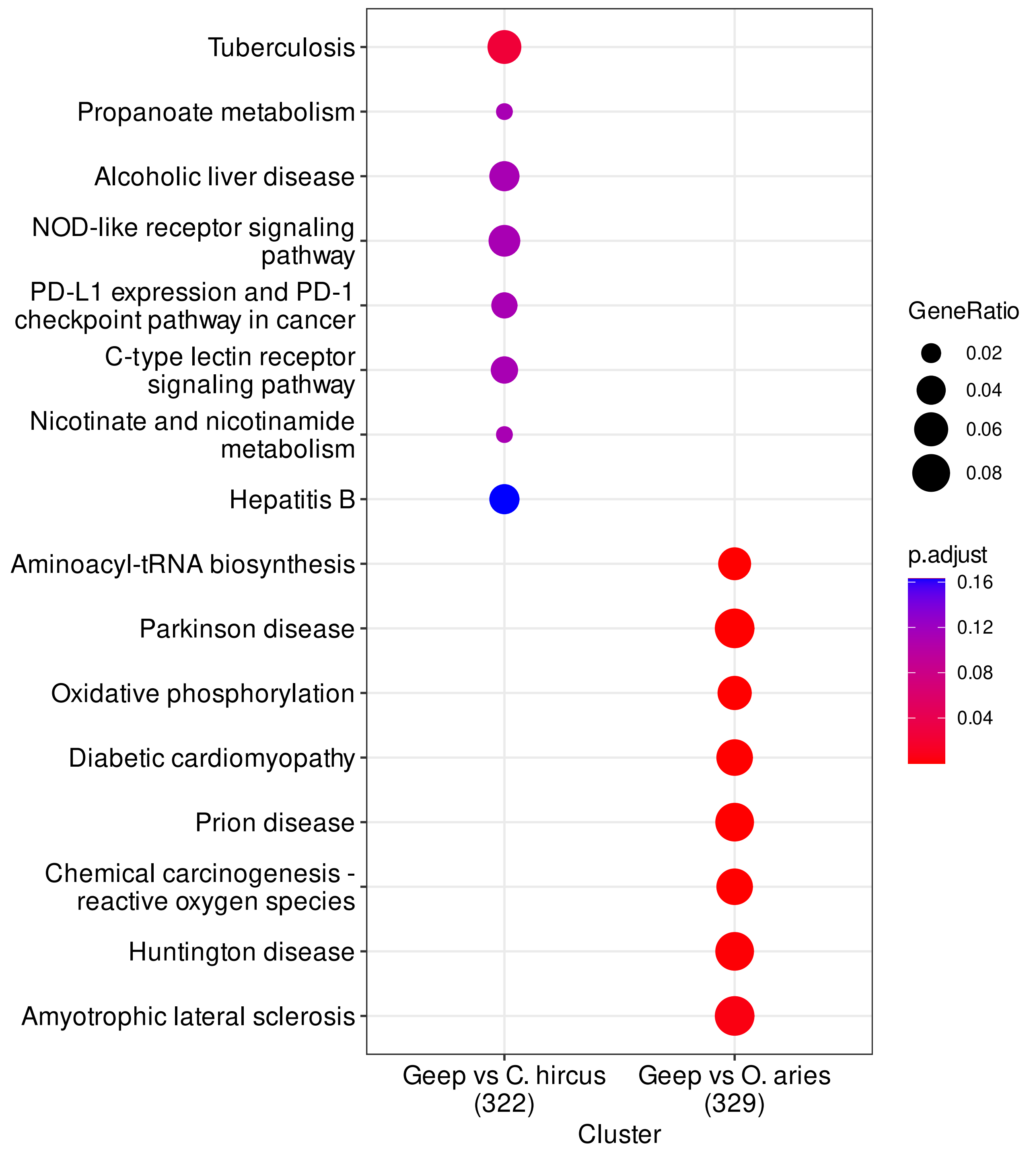

Supplement: Supplementary file 1 — Supplementary Information 1. [file 41598_2023_38407_MOESM1_ESM.pdf]
